# Effects of Antenatal Maternal Depression and Anxiety on Children’s Early Cognitive Development: A Prospective Cohort Study

**DOI:** 10.1371/journal.pone.0135849

**Published:** 2015-08-28

**Authors:** Gladys Ibanez, Jonathan Y. Bernard, Claire Rondet, Hugo Peyre, Anne Forhan, Monique Kaminski, Marie-Josèphe Saurel-Cubizolles, EDEN Mother-Child Cohort Study Group

**Affiliations:** 1 Paris-Descartes University, INSERM Obstetrical, Perinatal and Pediatric Epidemiology Research team, Center for Epidemiology and Biostatistics (U1153), Paris, France; 2 School of Medicine, Department of General Practice, UPMC University Paris 06, Paris, France; 3 INSERM, Center for Research in Epidemiology and Population Health (CESP), U1018, Team "Epidemiology of diabetes, obesity and kidney disease: lifelong approach", F-94807 Villejuif, France; 4 Univ Paris-Sud, UMRS 1018, F-94807, Villejuif, France; 5 Laboratoire de Sciences Cognitives et Psycholinguistique, Ecole Normale Supérieure, CNRS, EHESS, Paris, France; 6 Hôpital Robert Debré, Service de Psychopathologie de l'Enfant et de l'Adolescent, APHP, Paris, France; Hamamatsu University School of Medicine, JAPAN

## Abstract

**Introduction:**

Studies have shown that depression or anxiety occur in 10–20% of pregnant women. These disorders are often undertreated and may affect mothers and children’s health. This study investigates the relation between antenatal maternal depression, anxiety and children’s early cognitive development among 1380 two-year-old children and 1227 three-year-old children.

**Methods:**

In the French EDEN Mother-Child Cohort Study, language ability was assessed with the Communicative Development Inventory at 2 years of age and overall development with the Ages and Stages Questionnaire at 3 years of age. Multiple regressions and structural equation modeling were used to examine links between depression, anxiety during pregnancy and child cognitive development.

**Results:**

We found strong significant associations between maternal antenatal anxiety and poorer children’s cognitive development at 2 and 3 years. Antenatal maternal depression was not associated with child development, except when antenatal maternal anxiety was also present. Both postnatal maternal depression and parental stimulation appeared to play mediating roles in the relation between antenatal maternal anxiety and children’s cognitive development. At 3 years, parental stimulation mediated 13.2% of the effect of antenatal maternal anxiety while postnatal maternal depression mediated 26.5%.

**Discussion:**

The partial nature of these effects suggests that other mediators may play a role. Implications for theory and research on child development are discussed.

## Introduction

The fetal programming hypothesis, the Developmental Origins of Health and Disease paradigm (DOHaD) and the Developmental Origins of Behaviour, Health and Disease paradigm (DOBHaD) suggest that human health and development have their origin in early life [1, 2]. Central to these hypotheses is the interdependence of developmental influences, either genetic or environmental. Pregnancy and the early postnatal period are times of great vulnerability [3].

Approximately 10 percent of women suffer from depression during pregnancy, a rate that varies according to women’s individual histories, socioeconomic factors and exposure to proximal stressors [4–6]. Maternal depression, anxiety, and stress during pregnancy have been associated with poor fetal development and poor birth outcomes, including preterm birth (PTB) and low birth weight (LBW) [4, 7]. PTB or LBW children are at risk of emotional or cognitive problems, including an increased risk of attention deficit/hyperactivity, anxiety, or language delay [8]. Moreover, prenatal maternal stress results in early programming of brain functions with permanent changes in neuroendocrine regulation and behaviour in offspring [9]. These changes may affect cognitive and emotional processing of children. Hormonal dysregulation affecting the hypothalamus-pituitary-adrenal (HPA) axis or the autonomous nervous system may be involved in these associations [3, 10].

Many studies have investigated the effects of antenatal maternal depression or anxiety on children’s cognitive development. Some authors found that antenatal maternal depression was associated with poorer children’s cognitive development [11–13]. Other authors found that antenatal maternal anxiety was associated with delays in children’s cognitive development [14–17]. While depression and anxiety have both been associated with poor children’s cognitive development, there have been few attempts to compare their effects. Clark et al. proposed a tripartite model of anxiety and depression. This model suggests the presence of a common general distress, a physiological hyperarousal (specific anxiety) and an anhedonia (specific depression) [18]. Consequently, depression and anxiety may have differential effects on child development, even though they are highly associated. Moreover, these two conditions are important to distinct as they may require different types of interventions during pregnancy (relaxation, psychotherapy, antidepressants, anxiolytics, etc) [19].

In a review of the literature in 1999, Goodman and Gotlib described four mechanisms through which maternal risk might be transmitted to children [20]. These mechanisms could occur prenatally or postnatally: heritability of depression; dysfunctional neuroregulatory mechanisms; exposure to negative maternal cognitions, behaviors and affects; and stressful context of the children’s lives. As a result, postpartum factors such as maternal negative affects or inadequate parenting may be mediators of the relation between maternal prenatal depression or anxiety and child cognitive development. However, there are few studies establishing the respective roles of these variables as mediators of the association between maternal prenatal depression or anxiety and cognitive development of children.

The purpose of this study was to explore the relations between antenatal maternal depression, anxiety and their children’s early cognitive development in a large prospective cohort of pregnant women, by taking into account the role of potential mediating variables.

## Methods

### Study design

The EDEN study is a French cohort of prenatal and early postnatal determinants of child health and development. It includes all French-speaking women seeking prenatal care before the 24^th^ week of gestation at two maternity centers (in Nancy and Poitiers) between September 2003 and January 2006. Exclusion criteria were twin pregnancy, diabetes before pregnancy, and any plan to move out of the region in the next three years. Of the eligible women, 55% (2002 women) agreed to participate [21].

### Measures

#### Mental health

At 24–28 weeks of gestation, the women’s depressive symptoms were assessed by using the Center for Epidemiological Studies Depression scale (CES-D) and their anxious symptoms by using the State Trait Anxiety Inventory (the STAI state component). At 4, 8, and 12 months postpartum, maternal depressive and anxious symptoms were assessed by using the Edinburgh Postnatal Depression Scale (EPDS). At 3 years after birth, maternal depressive symptoms were again assessed by using the CES-D. All three questionnaires were self-administered.

The CES-D was developed by the US National Institute of Mental Health and was translated into French by Fuhrer and al [22, 23]. High internal consistency has been reported with Cronbach’s alpha coefficients ranging from 0.85 to 0.90 across studies. It contains 20 items reflecting major dimensions of depression. Response categories are scored on a 4-point scale ranging from 0 (rarely or never) to 3 (most or all of the time). Total scores range from 0 to 60. Following previous research, we considered a score of 16 or higher to detect a high level of depressive symptoms [24, 25]. Sensitivity analyses were also performed with a cut-off at 23 or higher, and when considering CES-D as a continuous variable.

The STAI was developed by Spielberger and al. in 1970 and was translated into French in 1993 [26, 27]. It measures two types of anxiety: state anxiety, which is the current state of anxiety, and trait anxiety which evaluates relatively stable aspects of anxiety. Internal consistency coefficients for the scales have ranged from .86 to .95. In this study, we used the State-STAI, which includes 20 items. Responses scored from 1 to 4 (1 corresponds to the lowest degree of anxiety, 4 to the highest degree). In the absence of a consensus in the literature, the threshold of 37 (≥37, i.e. the 80th percentile of our sample) was used to distinguish anxious from non-anxious women. Sensitivity analyses were also performed with a cut-off at 40 or higher, and when considering STAI as a continuous variable.

The analyses of depression and anxiety included all women who responded to at least 19 of the 20 items of the CES-D and the STAI. When only one item was missing, it was arbitrarily given the most favorable value, 0 for the CES-D and 1 for the STAI. The women were classified into four groups: non-depressed and non-anxious, depressed but non-anxious, anxious but non-depressed, and depressed and anxious women (in the text, the terms ‘depression’ and ‘anxiety’ are sometimes used as a short-hand for those women who scored above the specified cutoff on the CES-D or the STAI. However, both scales are not diagnostic tools).

The EPDS is a widely used 10-item questionnaire providing an indication of the mother’s mood in the year following birth. Developed by Cox et al., it was translated and validated in French by Guédeney and Fermanian in 1998 [28, 29]. Items are rated on a 4-point scale ranging from 0 (not at all) to 3 (most of the time). A score of 13 and higher is indicative of significant depressive symptoms [28].

#### Child development

The MacArthur Communicative Development Inventory (CDI) is a parent-report tool used to assess monolingual children’s vocabulary acquisition [30]. It has been validated in French in three short forms (12, 18, and 24 months of age) [31]. Here, we used it at the age of 24 months ± 2 months. Parents report the words that the child uses spontaneously from a list of 100. CDI scores thus range from 0 to 100 and were analyzed both as means and percentages of children below the 15th percentile of the distribution according to previous research [32].

The Ages and Stages Questionnaire (ASQ) is a parent-completed questionnaire aimed at assessing the developmental status of infants and young children [33]. Questionnaires are available for 2-months intervals from 4 to 24 months and then for 30, 33, 36, 48, 54, and 60 months. Here, we used it at the age of 36 months ± 2 months. The ASQ includes six questions in each of five areas (communication, gross motor skills, fine motor skills, problem solving, and personal-social skills). Each question can be answered: yes (the child can perform the task requested: 10 points), sometimes (the child can sometimes perform the task requested: 5 points) and not yet (the child is not yet able to perform the task: 0 points). Area scores range from 0 to 60 points, and total ASQ scores from 0 to 300 points. ASQ scores were used both as means and percentages of children below the cutoff scores established in the US population (mean score minus two standard deviations) [34, 35].

#### Other characteristics

Women’s characteristics were obtained by an interview at 24–28 weeks of pregnancy. These data included mother's age, educational level, household income, couple situation, parity, smoking habits during pregnancy, and family history of language delay. The child’s gestational age at delivery, birth weight, gender, and breastfeeding status during the hospital stay and at discharge were obtained from medical records. Preterm birth was defined as birth before 37 completed weeks of gestation. After birth, questionnaires were sent to families at 4, 8, 12, 24, and 36 months. The frequency of parental stimulation was defined as weekly frequency of storytelling, singing, and playing with the child by both parents, reported at 2 and 3 years. By averaging this frequency at 2 years of age, the frequency of parental stimulation was estimated on a scale that ranged from 1 (shared activities less than once a week) to 5 (shared activities nearly every day). In the 2-years questionnaire, mothers reported the child’s daytime caretaker: mother, family (father, grandparents), creche (daycare center) or other (paid child carer, or neighbor). At 3 years, mothers reported whether the child had started school (free but not mandatory public education begins in France at age 3).

### Ethics

This study was approved by the Ethics Committee of Bicêtre Hospital (CCPRB) and the National Commission on Data Processing and Liberties (CNIL). All women consented in writing to participate in the study with their infant.

### Statistical analysis

Women’s mental health and other antenatal and children’s characteristics were described with descriptive statistics. Then, reliability of the mental health questionnaires was evaluated. We looked at associations between antenatal maternal depression and anxiety, and postnatal factors assumed to influence children’s cognitive development: breastfeeding, postnatal maternal depression, frequency of parental stimulation at 2 and 3 years, family history of language delay, main caretaker at 2 years, and pre-elementary school at 3 years. We used both bivariate and multivariate analyses to assess the relations between antenatal maternal depression and anxiety and the children’s cognitive assessment scores at 2 and 3 years. To test the association between antenatal maternal depression and anxiety and developmental scores at 2 years and 3 years, the following potential confounders were considered: mother’s age, maternal educational level, household income, couple situation, parity, smoking during pregnancy, gestational age, birth weight, sex, breastfeeding during the hospital maternity stay, postnatal maternal depressive symptoms at 4, 8, 12 months or 3 years after birth, postnatal parental stimulation, caretaker and maternity units. Four successive models were proposed: an unadjusted model (model A1), a model including the confounding variables mentioned above except for postnatal maternal depression and postpartum parental stimulation (model A2), a model with these confounding variables and postnatal maternal depression (model A3) and a model with these confounding variables including postnatal maternal depression and postnatal parental stimulation (model A4). In the ASQ analyses for 3-year olds, we also adjusted for pre-elementary school attendance.

**Fig 1:**
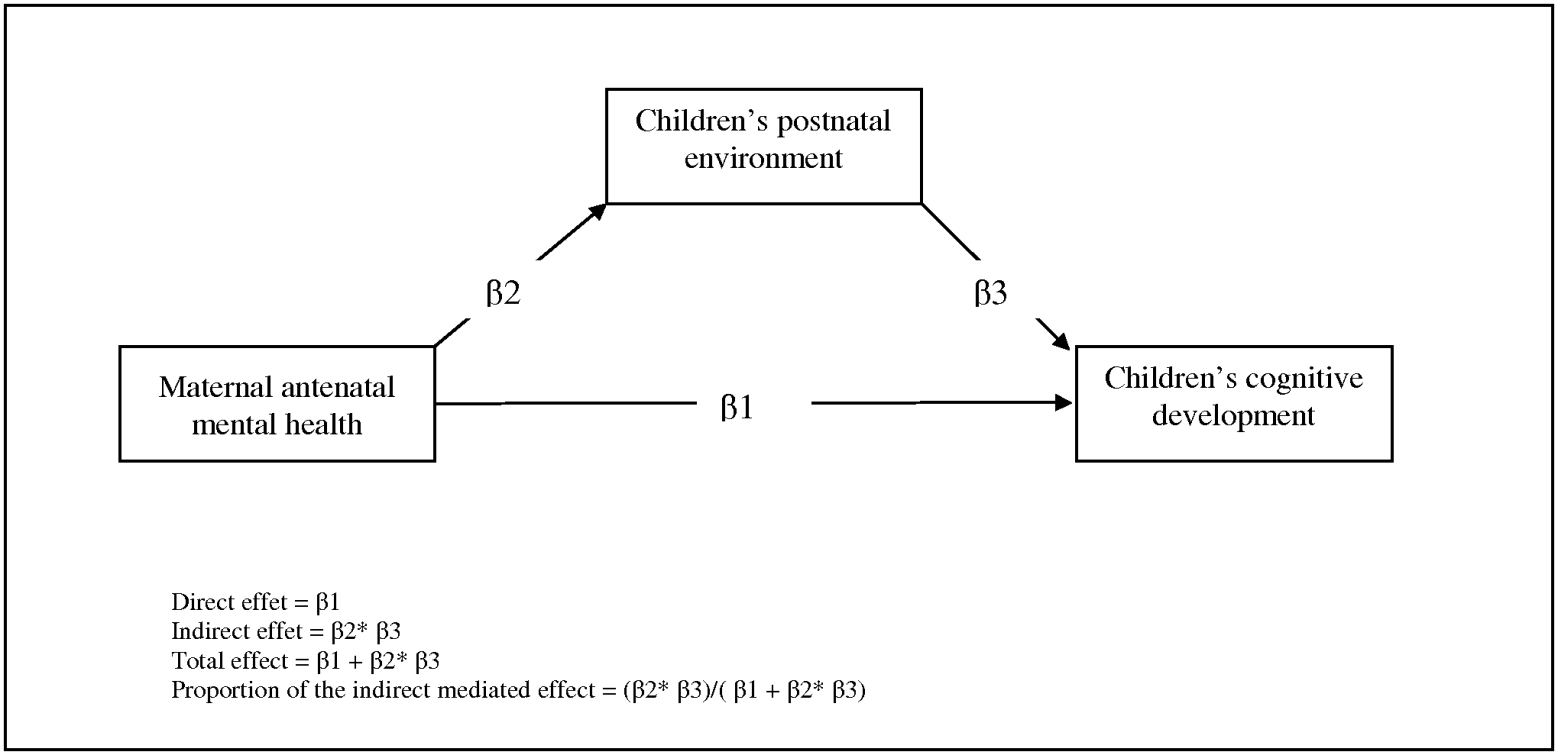
Structural equation model: Mediating model of the association between maternal antenatal mental health and impaired children’s cognitive development.

We used chi-square and ANOVA tests for bivariate analyses. The internal consistency of the CES-D and the STAI questionnaires was estimated by applying Cronbach’s alpha coefficient. The Spearman correlation was used to quantify the association between the two mental health assessment scores and the two cognitive assessment scores. Logistic or linear regression models were used for multivariate analyses. Lastly, structural equation modeling was used to estimate the mediated effects of postnatal maternal depression and parental stimulation in the relation between antenatal maternal anxiety and cognitive development at 2 and 3 years (Fig 1). Structural equation models are a generalization of linear regression and factor analysis models [36]. In this model, CDI and total ASQ scores were included as continuous variables. We made the hypothesis of non independent covariances between the variables “parental stimulation” and “postnatal maternal depression”. The root mean square error of approximation (RMSEA) and the comparative fit index (CFI) were used to assess each model’s fit. Fit was considered to be good when the RMSEA was lower than 0.05 and the CFI greater than 0.95. P value were considered significant when *p* <0.05. Data were analysed with SAS 9.2 software.

## Results

### Depression and anxiety during pregnancy

**Fig 2:**
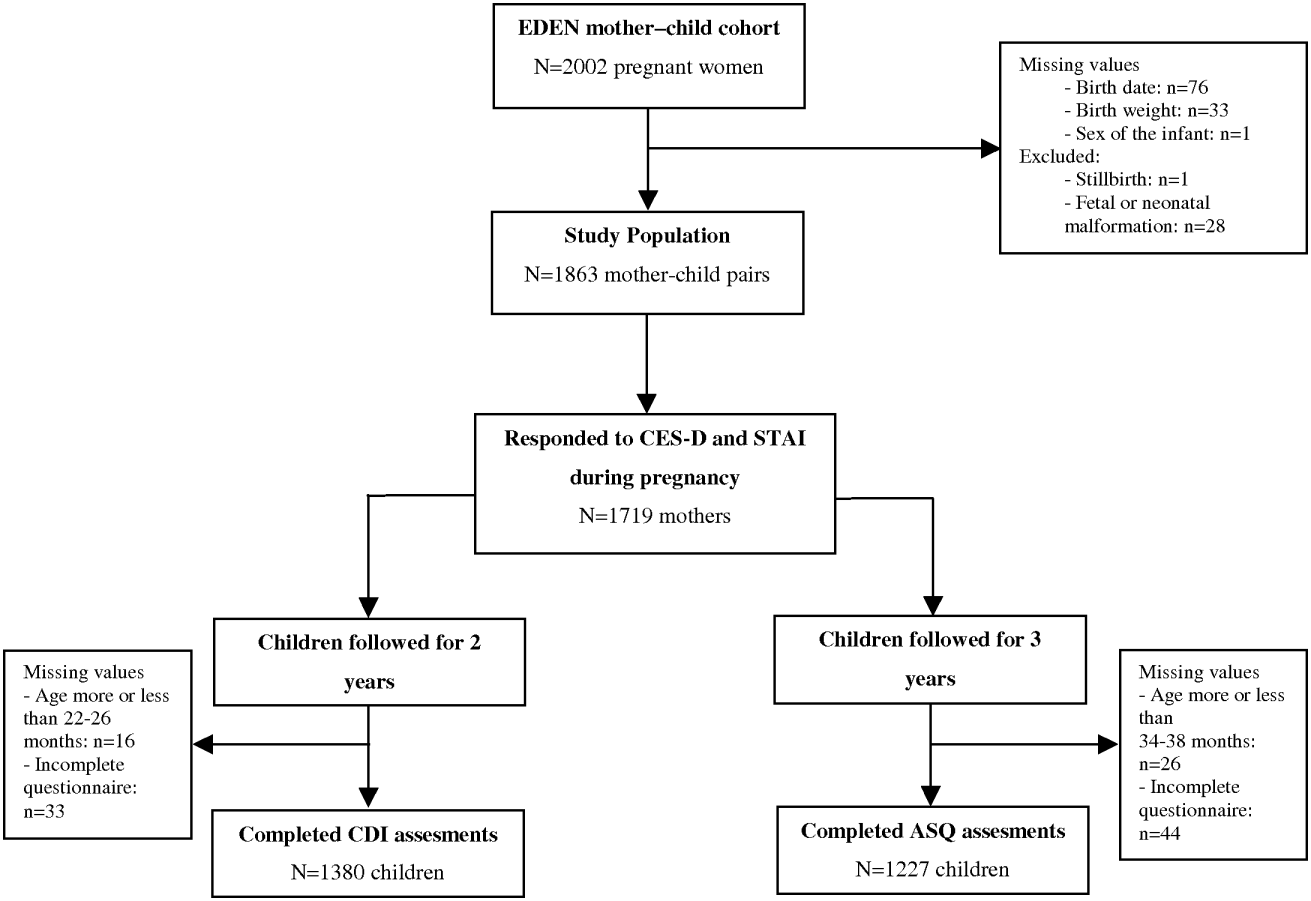
Flow chart of children assessed with the CDI at 2 years of age or the ASQ at 3 years of age.

Data for the initial analysis were available for 1863 mother-infant pairs of the 2002 women recruited for the EDEN cohort (Fig 2). Of the women who responded to both the CES-D and STAI questionnaires (n = 1719), 7.9% (n = 135) were classified as anxious only, 11.8% (n = 203) as depressed only, and 13.2% (n = 227) as depressed and anxious. The CES-D and STAI questionnaires appeared to have a high internal consistency (Cronbach's α coefficient = 0.95 and 0.91 respectively). The Spearman correlation coefficient between the CES-D and STAI scores was 0.25 (*p* < 0.0001).

Compared to women who responded to both questionnaires, those with one unknown score (for depressed or anxious symptoms) had a higher score on the other mental health scale and a lower educational level [37]. Fourteen (0.7%) women did not complete the self-administered questionnaire at all. Women classified as depressed and anxious had higher mean CES-D scores than those classified only as depressed and higher mean STAI scores than women classified only as anxious. Compared to women who were neither anxious nor depressed, women who were anxious or depressed had a lower educational level, lower household income, and lived alone more often. Depressed women were also more often multiparous, and anxious women smoked more often during pregnancy.

We compared mother–child pairs included and not included (due to attrition and exclusions) in the analysis: At 2 and 3 years of age, included mothers were significantly older, more often primiparous and smoker than non included mothers and had a higher level of education (p<0.05). Children’s characteristics did not differ between those included and not included in the analysis.

Women’s antenatal mental health was not significantly associated with preterm birth. Children of mothers classified as depressed and anxious were breastfed less often than those of women classified as non depressed and non anxious (Table 1). In the postnatal period, 19.0% of all mothers experienced symptoms of depression at least once within the first year and 27.6% symptoms at least once between birth and 3 years. Women who were anxious or depressed during pregnancy had postnatal symptoms of depression more often than those who were neither anxious nor depressed (Table 1). Parental stimulation at 3 years after birth was less common for women classified as depressed and anxious during pregnancy. The groups did not differ in family history of language delay. The children of women depressed during pregnancy were in daycare more often than the others. Finally, pre-elementary schooling did not differ according to whether women were depressed or anxious during pregnancy.

**Table 1:** Characteristics of parents and children according to mothers’ mental health during pregnancy.

Characteristics	Non depressive, non anxious N = 1154	Anxious, non depressive N = 135		Depressive, non anxious N = 203		Depressive and anxious N = 227		Global *p*-value^2^
	m± sd or % (n)	m± sd or % (n)	*p* ^1^	m± sd or % (n)	*p* ^1^	m± sd or % (n)	*p* ^1^	
**Child at birth**								
Gestational age, weeks	39.3 ± 1.6	39.1 ± 2.1	0.19	39.3 ± 1.8	0.63	39.1 ± 1.9	0.06	0.19
Preterm birth	5.0 (58)	5.9 (8)	0.65	5.9 (12)	0.60	7.5 (17)	0.13	0.51
Birth weight, g	3278± 484	3256 ± 540	0.97	3358 ± 531	0.23	3252 ± 548	0.92	0.45
**Parents’ social environment**								
Maternal age, years								
<25	14.5	17.0		19.2		18.5		
[25–35]	70.3	68.2	0.73	65.0	0.19	63.4	0.11	0.34
≥ 35	15.2	14.8		15.8		18.1		
Household income, euros								
0–1500	12.8	20.7		19.2		24.2		
1501–3000	56.7	52.6	0.04	60.1	0.004	52.0	<0.0001	<0.0001
3001 and more	30.5	26.7		20.7		23.8		
Educational level								
No degree	24.0	35.8	0.03	27.1	0.01	36.6	<0.0001	0.01
End of secondary school	17.4	16.4		25.6		16.9		
≤2 years university degree	23.1	17.9		19.6		24.1		
≥3 years university degree	35.5	29.9		27.7		22.3		
Partner status								
Married	55.4	51.9	0.04	50.7	<0.0001	46.2	<0.0001	<0.0001
Unmarried, living with a partner	41.0	40.0		38.5		42.2		
Single	3.6	8.1		10.8		11.6		
**Parity**								
Primiparous	47.8	46.7	0.81	39.1	0.02	38.9	0.02	0.03
**Family history of language delay**	6.2	6.0	0.92	7.9	0.36	5.7	0.79	0.79
**Breastfeeding initiation**	73.5	67.2	0.12	77.8	0.19	64.8	0.008	0.007
**Postnatal maternal depression**								
Depressive symptoms until 1 year after birth	12.8	29.0	<0.0001	36.8	<0.0001	50.0	<0.0001	<0.0001
Depressive symptoms until 3 years after birth	11.3	24.8	<0.0001	30.5	<0.0001	40.7	<0.0001	<0.0001
**Child’s environment**								
Frequency of parental stimulation at 2 years								
≤ 1-2/w	4.3	7.2	0.40	4.8	0.10	4.5	0.50	0.29
3-6/w	15.0	13.4		21.9		18.7		
7/w	80.6	79.4		73.3		76.8		
Frequency of parental stimulation at 3 years								
≤ 1-2/w	4.0	3.3	0.28	6.5	0.16	8.9	0.04	0.08
3-6/w	9.1	14.1		12.9		9.6		
7/w	86.9	82.6		80.7		81.5		
Caretaker at 2 years								
Mother	37.9	46.7	0.15	49.3	0.009	52.0	0.0008	0.0009
Family	8.2	5.2		8.9		7.5		
Nursery	18.5	19.3		30.1		28.6		
Other	35.4	28.9		11.8		11.9		
Attended pre-elementary school at 3 years	65.9	65.2	0.90	71.0	0.26	69.1	0.46	0.63

^1^ Statistical comparison of this group with the “non depressed, non anxious” women (first column)

^2^ Global *p* value of chi-square test or ANOVA test.

### Cognitive development at 2 and 3 years

In our sample, 1380 children were assessed at 2 years with the CDI and 1227 children at 3 years with the ASQ (Fig 2). Children not assessed at these ages had mothers who were younger and more often socially disadvantaged, multiparous, and smokers. Mean CDI scores were 60.6 ± 29.5 (mean ± SD) and ASQ scores 269.5 ± 30.1. The Spearman correlation coefficient between the CDI and ASQ scores was 0.44 (*p* < 0.0001). The percentage of children below the critical threshold was 14.1% for the CDI at 2 years and 15.7% for the ASQ at 3 years.

In bivariate analyses, children whose mothers were classified as only anxious during pregnancy were associated to higher rates of abnormal CDI or total ASQ scores (Table 2-Model A1). The main domains affected were communication, gross motor and fine motor (Table 3). Antenatal maternal depression was not associated with child development, except for the gross motor domain and only when also associated with antenatal anxiety. Results were similar when cut-off scores were respectively 23 and 40 for the CES-D and the STAI: children whose mothers were classified as only anxious during pregnancy were associated to higher rates of abnormal CDI or total ASQ scores with adjusted odds ratios of 1.70 (95% CI, 1.06–2.73) and 1.78 (95% CI, 1.14–2.79) respectively. When CES-D and STAI scores were treated as continuous variables, maternal anxiety was negatively and significantly associated with the total ASQ scores in the adjusted model (b^total ASQ^ = -0.29, 95% CI:-0.40;-0.11, *p* = 0.002). Maternal anxiety was not associated with CDI scores (b^CDI^ = -0.09, 95% CI:-0.27;+0.09, *p* = 0.34). Maternal depression was not associated with CDI or total ASQ scores: b^CDI^ = -0.02 (95% CI:-0.23;+0.20, *p* = 0.89) and b^total ASQ^ = -0.11 (95% CI: -0.35;+0.13, *p* = 0.35) respectively.

**Table 2:** Depression, anxiety, and children cognitive scores at 2 years and 3 years (unadjusted and adjusted analyses).

		Non depressive, non anxious	Anxious, non depressive		Depressive, non anxious		Depressive and anxious		*p*-value^6^
		% or OR [CI 95%]	% or OR [CI 95%]	*p* ^5^	% or OR [CI 95%]	*p* ^5^	% or OR [CI 95%]	*p* ^5^	
**CDI < threshold**	**Model A1** 1 (N = 1268)	13.5	25.6	0.002	10.4	0.30	13.6	0.98	0.009
	**Model A2** 2 (N = 1218)	1	2.13 [1.22–3.74]	0.008	0.60 [0.32–1.12]	0.11	0.86 [0.49–1.50]	0.60	0.01
	**Model A3** 3 (N = 1118)	1	1.94 [1.07–3.52]	0.03	0.64 [0.33–1.24]	0.19	0.90 [0.49–1.63]	0.72	0.05
	**Model A4** 4 (N = 1107)	1	1.94 [1.06–3.53]	0.03	0.63 [0.33–1.22]	0.17	0.90 [0.50–1.65]	0.74	0.05
**Total ASQ < threshold**	**Model A1** 1 (N = 1130)	14.6	25.8	0.006	11.8	0.41	18.9	0.23	0.02
	**Model A2** ^2^ (N = 1084)	1	2.03 [1.17–3.52]	0.01	0.77 [0.41–1.44]	0.41	1.37 [0.82–2.30]	0.21	0.03
	**Model A3** ^3^ (N = 1079)	1	1.93 [1.11–3.37]	0.02	0.69 [0.36–1.31]	0.26	1.21 [0.70–2.10]	0.48	0.05
	**Model A4** ^4^ (N = 1060)	1	2.05 [1.17–3.60]	0.01	0.61 [0.31–1.20]	0.15	1.16 [0.66–2.02]	0.69	0.02

^1^ The model A1 is unadjusted

^2^ The model A2 is adjusted for: mother’s age, maternal educational level, household income, couple situation, parity, smoking during pregnancy, gestational age, birth weight, sex, breastfeeding, caretaker and center

^3^ The model A3 is adjusted for the same variables than A2 + maternal depression

^4^ The model A4 is adjusted for the same variables than A3 + postnatal parental stimulation

^5^ Statistical comparison of this group with the “non depressed, non anxious” women (first column)

^6^ Global *p* value of chi-square test for model A1 or Wald test for models A2-A4.

**Table 3:** Depression, anxiety, and score for each domain on the ASQ at 3 years (unadjusted and adjusted analyses).

		Non depressive, non anxious	Anxious, non depressive		Depressive, non anxious		Depressive and anxious		*p*-value^3^
		% or OR [CI 95%]	% or OR [CI 95%]	*p* ^2^	% or OR [CI 95%]	*p* ^2^	% or OR [CI 95%]	*p* ^2^	
	**Communication**	2.6	7.9	0.008	2.5	0.94	5.5	0.08	0.03
		1	3.14 [1.30–7.62]		0.85 [0.28–3.24]		2.13 [0.89–5.12]		
	**Gross motor**	3.9	5.6	0.44	3.4	0.77	10.2	0.002	0.02
**ASQ < total threshold,**		1	1.47 [0.56–3.87]		0.86 [0.30–2.47]		2.78 |1.41–5.47]		
	**Fine motor**	4.8	15.7	<0.0001	5.0	0.90	3.9	0.66	0.003
Unadjusted analyses		1	3.71 [1.93–7.16]		1.06 [0.44–2.56]		0.81 [0.31–2.10]		
N = 1130	**Problem solving**	6.2	5.6	0.84	5.9	0.90	9.4	0.18	0.56
		1	0.91 [0.35–2.33]		0.95 [0.42–2.15]		1.57 [0.81–3.05]		
	**Personal social**	2.5	5.6	0.09	4.2	0.29	3.9	0.40	0.32
		1	2.30 [0.84–6.30]		1.70 [0.63–4.61]		1.57 [0.58–4.27]		
	**Communication**	1	2.58 [0.93–7.22]	0.07	0.61 [0.13–2.81]	0.52	1.52 [0.50–4.58]	0.46	0.23
**ASQ < total threshold,**	**Gross motor**	1	1.60 [0.59–4.35]	0.36	0.71 [0.23–2.17]	0.55	2.28 [1.06–4.93]	0.04	0.11
	**Fine motor**	1	4.38 [1.97–9.71]	0.0003	1.18 [0.41–3.39]	0.77	0.61 [0.19–2.01]	0.42	0.001
Adjusted analyses^1^									
N = 1060	**Problem solving**	1	0.93 [0.35–2.51]	0.89	0.56 [0.20–1.53]	0.26	1.30 [0.60–2.82]	0.51	0.55
	**Personal social**	1	2.76 [0.80–8.69]	0.08	1.22 [0.32–4.64]	0.77	1.69 [0.48–5.93]	0.41	0.37

^1^ The model is adjusted for: mother’s age, maternal educational level, household income, couple situation, parity, smoking during pregnancy, gestational age, birth weight, sex, breastfeeding, caretaker, maternal depression, postnatal parental stimulation and center

^2^ Statistical comparison of this group with the “non depressed, non anxious” women (first column)

^3^ Global p value of chi-square test for unadjusted analyses or Wald test for adjusted analyses.

**Fig 3:**
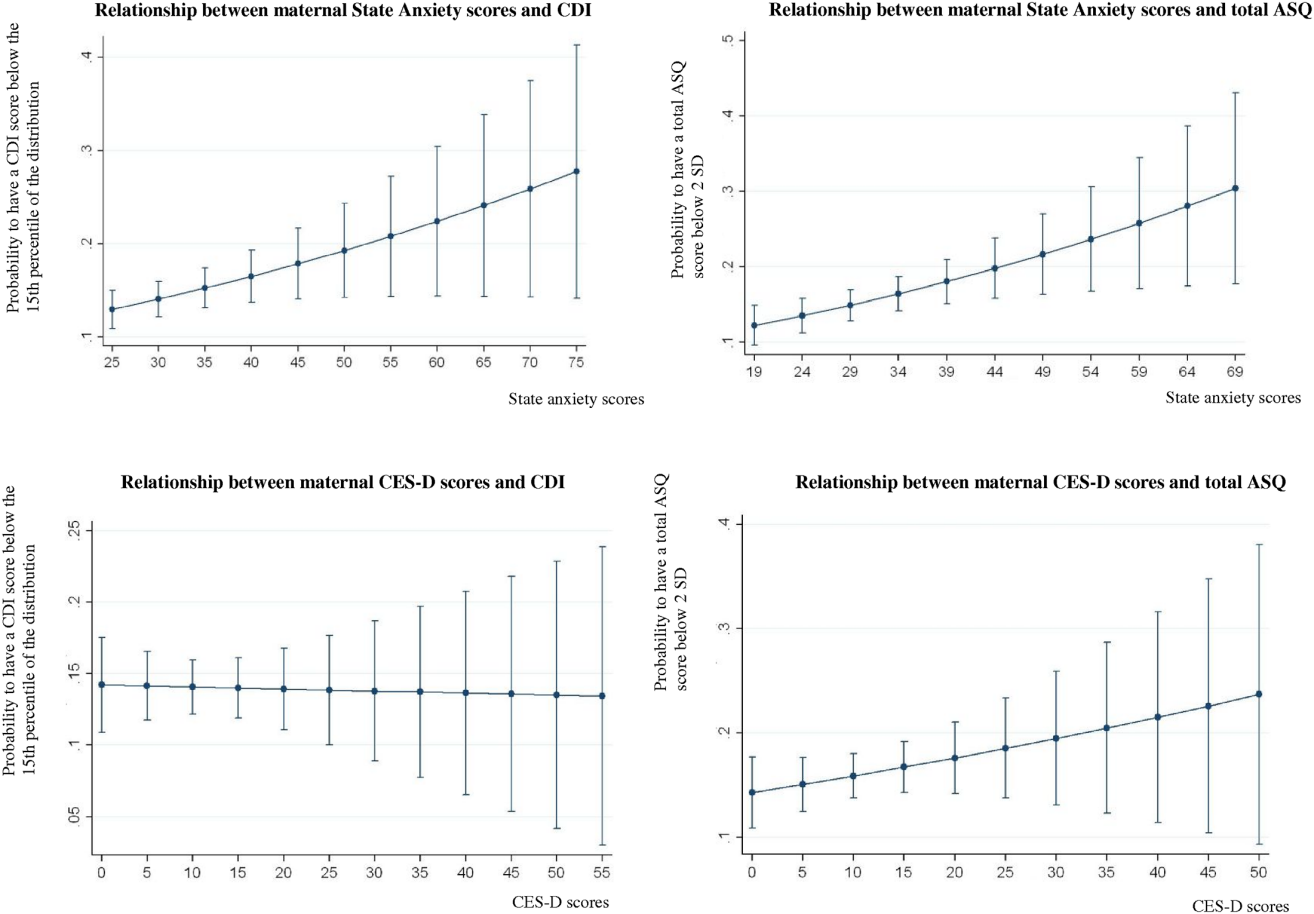
Relationship between anxiety or depression scores and children cognitive development in the EDEN cohort—Prediction models (bivariate analyses).

Inter-relations between anxiety or depression scores and the percentage of developmental scores below the threshold are shown in Fig 3. At 2 years, data do not suggest a relationship between depression or anxiety scores and CDI scores. At 3 years, data suggest a dose-response relationship between anxiety scores and total ASQ scores. There was no relationship between depression scores and total ASQ scores.

After adjustments, the children of anxious women during pregnancy remained associated to higher rates of abnormal CDI or total ASQ scores. The main domain affected was the fine motor, with an odds ratio equal to 4.38 [1.97–9.71] (Table 2-Model A2-A4). The children of depressed and anxious women during pregnancy had higher rates of abnormal ASQ scores in the gross motor domain. Antenatal maternal depression alone was not associated with child cognitive development.

**Fig 4:**
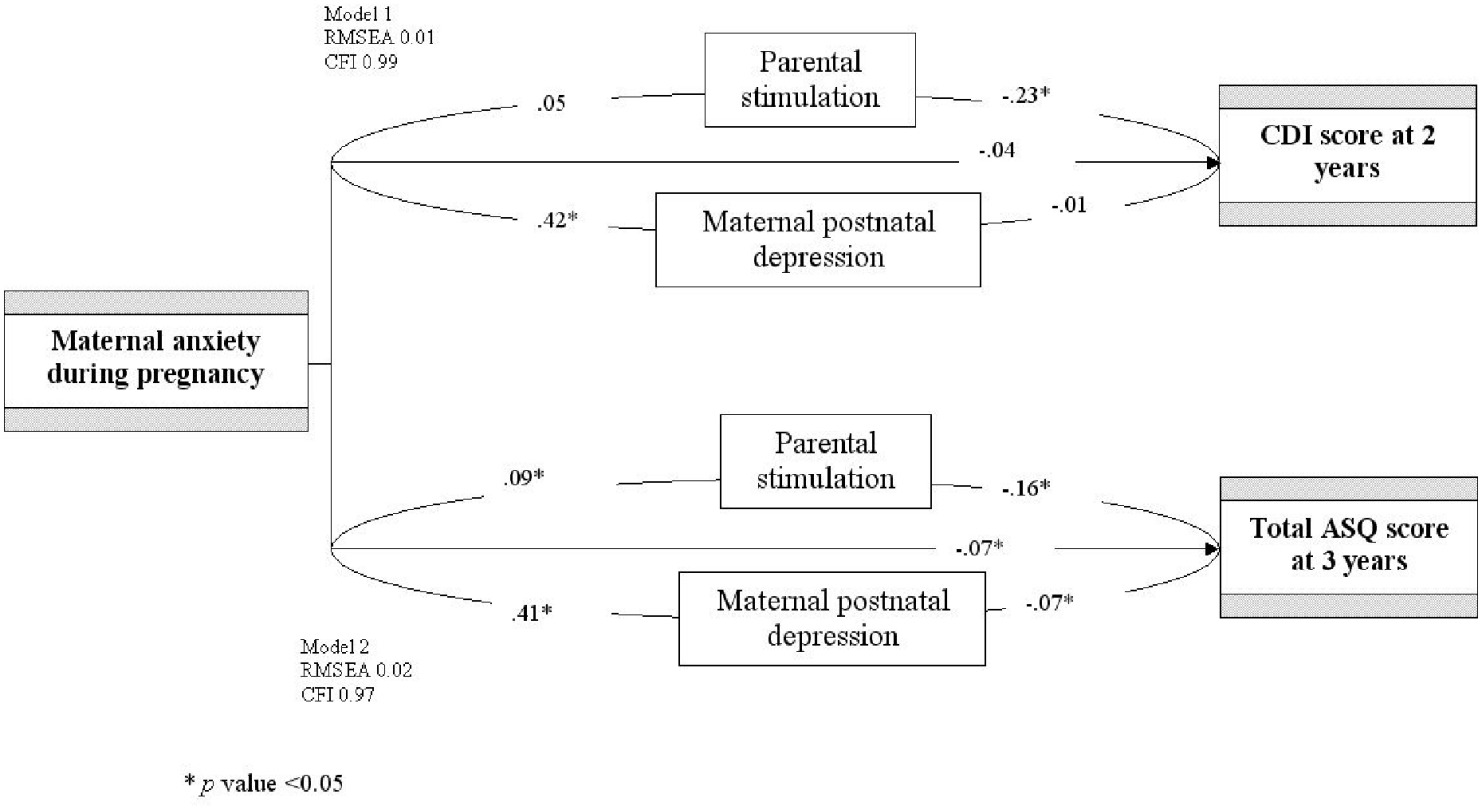
Results of the structural equation models: Mediating effects in the association between antenatal maternal anxiety and children’s cognitive development at 2 and 3 years.

In a structural equation model, CDI and ASQ scores at 2 or 3 years were strongly associated with antenatal maternal anxiety symptoms (Fig 4). When the postnatal maternal depressive symptoms or the parental stimulation were added to the equation as mediating variables, associations between antenatal maternal anxiety symptoms and CDI scores were at the limit of significance (t value = 1.63, *p* = 0.06). When both potential mediators were included in the model (with the hypothesis of non independant covariances between them), the straight arrow was no longer significant (t value = 1.33, *p* = 0.08).

At 3 years, all paths were significant between antenatal maternal anxiety symptoms, postnatal maternal depressive symptoms, parental stimulation and ASQ scores. Parental stimulation mediated 13.2% of the effect of maternal antenatal anxiety while postnatal maternal depression mediated 26.5%. Mediation by each of these factors was weaker than the direct effect of antenatal maternal anxiety. The fit of each model was good, since the RMSEA was lower than 0.05 and the CFI greater than 0.95.

DiscussionThe study explored the association between depression, anxiety and child cognitive development. At two years, we found significant associations between maternal antenatal anxiety and poorer children’s cognitive development. However, beyond the mediating effects of postnatal maternal depression and parental stimulation, no significant direct effect was observed. At three years, we found significant associations between maternal antenatal anxiety and poorer children’s cognitive development in regression models and structural equation models. Both postnatal maternal depression and parental stimulation appeared to play mediating roles in the relation between antenatal maternal anxiety and children’s cognitive development. Mediation by each of these factors was weaker than the direct effect of antenatal maternal anxiety. The partial nature of these effects suggests that other mediators may play a role. Antenatal maternal depression was not associated with child development, except when antenatal maternal anxiety was also present.

To our knowledge, very few studies described the association of antenatal maternal depressive and anxious symptoms with children’s early cognitive development. We used a large prospective design and recruited mothers in two maternity units during pregnancy. The two child development measures were complementary: CDI specifically evaluates language ability, while ASQ tests other aspects of cognitive development [38]. This study combined two methodological approaches. A conceptual framework was used to manage the intricate inter-relations between antenatal maternal anxiety, postnatal depression, and parent-child interactions. Moreover, we controlled for many potential confounding variables during the analysis.

This study has several limitations. One is the possible bias in the sample selection: this sample of volunteers from 2 maternity centers is not representative of the general population. The sample is more socially advantaged than the general population, and this difference was further reinforced by the higher rate of loss to follow-up among the poorer and less educated families (attrition up to 20%). Additionally, this study considered state anxiety, although it might have been useful to examine the outcome measures according to the trait measure instead. Trait anxiety refers to a disposition or proneness to react with anxiety, while state anxiety refers to a transient emotional state, characterized by subjectively experienced tension and apprehension [15]. We used prenatal state anxiety in order to provide a valid measure of the intensity of transitory state anxiety subscale. In our study, the percentage of anxious women only (7.9%) was lesser than that of depressed women only (11.8%). The validity of the scales construct can be questioned. The cut-off score on the STAI was the 80^th^ percentile of the distribution, as there is no clinical cut-off validated in the literature. We performed the same analyses with different cut-offs for the STAI and the CES-D, with both variables treated as continuous. Finally, analyses were also performed with CDI and total ASQ scores as continuous variables. Similar results were observed especially with global development at 3 years. Furthermore, the CDI and ASQ are questionnaires completed by parents, rather than an assessment made directly by professionals during an examination. There is the potential report bias, i.e depressed mothers not recognising their children’s abilities [11]. Another limitation is that our analysis performed multiple comparisons, especially in each subscale of the ASQ. Statistical significance of p-values around 0.05 may be questioned. Anxious mothers were associated with impairment in the fine motor domain but not in the gross motor domain (adjusted *p* value = 0.11). However, anxious and depressed mothers were significantly associated with impairment in the gross motor domain. This may be due to a lack of power in our analyses (as we noted a tendancy with only anxious women) or to real differential effects. The role of comorbid maternal depression should be further explored in other studies. Then, we did not consider other mediators or potential risk factors for child cognitive development such as parental health literacy, geographical origins, marital conflicts or paternal depression [39, 40, 15]. Finally, our analysis had limited power to detect modest effects, especially those related to antenatal maternal depression.

Our findings are consistent with earlier studies that suggested an association between antenatal maternal anxiety and developmental impairment in children at 1, 2, 5 years as well as in adolescence [14–17]. In the literature, most studies exploring anxiety used the state-form of the STAI. Brouwers et al. found an association between high maternal anxiety levels (state and/or trait anxiety scores) and lower mental developmental scores at the age of two years. Van den Bergh et al., Mennes et al. and Loomans et al. only used the state-form of the STAI [15–17]. These authors found an association between prenatal maternal anxiety and children’s early cognitive development. Unfortunately, these studies did not consider both antenatal maternal depression and anxiety making these comparisons limited. Other studies found that antenatal maternal anxiety (but not antenatal maternal depression) was associated with behavioral or emotional problems in toddlers [41, 42, 10, 15]. Some studies have described an association between antenatal maternal depression and children’s cognitive development [11–13, 43]. In our study, the association between maternal antenatal anxious symptoms and child cognitive development was stronger in the absence of antenatal maternal depressive symptoms. These results may be due to chance, to insufficient power to detect an effect, or to other mediators not monitored in the study. Indeed, some authors have suggested that the effect of antenatal maternal psychological distress might be moderated by other prenatal factors (including maternal positive affects) and have described positive linear associations as well. Keim et al. found that moderate levels of antenatal maternal depression enhanced development among children exposed in utero [44]. DiPietro et al. found similar results with moderate levels of maternal psychological distress [45].

This study suggests that antenatal maternal anxiety has an influence on children’s subsequent cognitive development through maternal postnatal depression and parental stimulation. Postnatal maternal depression is known to be highly correlated with a lower sense of parenting efficacy [46–48]. Moreover, parents with depression reported a higher rate of conflicts events with their partners after the birth [49]. Antenatal maternal depression may be associated with marital conflicts, which are associated with poor child development [50]. Then, maternal and paternal depression are strongly correlated. Part of the effect of antenatal maternal depression on child cognitive development may be mediated by paternal depression [51]. In our study, preterm birth was not a significant mediator in the relation between antenatal maternal depression and child cognitive development.

In conclusion, there are accumulating evidences of association between antenatal maternal anxiety and specific cognitive functions in the offspring. We found that the standard confounders and mediators, including postnatal maternal mental health and parental stimulation, partially explained the effect of antenatal anxiety. These data could provide useful information to improve our understanding of the impact of antenatal depression and anxiety on children’s development. However, care in the interpretation is needed due to possible biases. It highlights the need to further research whether links between antenatal anxiety and cognitive abilities in offspring remain when objective measures of mental health or cognitive ability are used.
